# Analysis of short-term variation and long-term drift during reagent kit lot change in an NABL accredited clinical biochemistry laboratory

**DOI:** 10.5937/jomb0-25597

**Published:** 2021-01-26

**Authors:** Vivek Ambade, Pratibha Misra, Yaongamphi Vashum, Mukul Sharma, Bhasker Mukherjee, Kapil Bhatia, Manoj Puliyath, Ponnaiah Rasu, Prakash Berthwal Indra, Madathan Kandi Sibin

**Affiliations:** 1 Armed Forces Medical College, Department of Biochemistry, Pune, Maharashtra, India

**Keywords:** reagent lot change, quality control, clinical chemistry, long term drift, short term variation, NABL, NABL, kratkoročne varijacije, dugoročne promene, klinička hemija, kontrola kvaliteta, promena setova reagenasa

## Abstract

**Background:**

Kit lot change in clinical biochemistry labs leads to variations in patient results. This study planned to identify variations during 60 reagent lot changes in our laboratory during the period from June 2018 to May 2019.

**Methods:**

A statistical analysis was performed to identify the difference between patient samples results variations and QC results. The long term drift was analyzed using a regression test.

**Results:**

There was a significant difference between the patient and QC results in 16.7% of reagent lot changes. Moreover, the extent of variation in QC results was 3.3%. No long-term drift was seen in three analytes which were studied using regression analysis.

**Conclusions:**

Our results showed that, during reagent kit lot change, along with QC material, the patient samples should also be run in order to identify the variation. However, this practice is presently ignored by most of the laboratories. There was no accumulated effect in our laboratory due to reagent kit lot change.

## Abstract (sr)

**Uvod:**

Promena setova reagenasa u biohemijskim laboratorijama na klinikama dovodi do varijacija u rezultatima pacijenata. Plan ovog istraživanja je bio da identifikuje varijacije u toku šezdeset promena setova reagenasa u našoj laboratoriji u periodu od juna 2018. godine do maja 2019. godine.

**Metode:**

Izvršena je statistička analiza kako bi se identifikovala razlika između varijacija u rezultatima pacijenata i rezultata kontrole kvaliteta (QC). Dugoročne promene su analizirane pomoću regresijskih testova.

**Rezultati:**

Otkrivena je značajna razlika u rezultatima pacijenata i kontrole kvaliteta u 16,7% promena setova reagenasa. Takođe, opseg varijacije u rezultatima kontrole kvaliteta bio je 3,3%. Nisu pronađene dugoročne promene u tri analita koja su analizirana regresijskim testovima.

**Zaključak:**

Naši rezultati ukazuju na to da bi prilikom zamene setova reagenasa, pored materijala za kontrolu kvaliteta, trebalo testirati i uzorke pacijenata kako bi se identifikovale varijacije. Međutim, većina laboratorija ovaj postupak ne primenjuje. Nije pronađen dugoročni uticaj usled nagomilavanja varijacija prilikom promene setova reagenasa u našoj laboratoriji.

## Introduction

Delivering consistent and reliable test results is the primary objective of a clinical chemistry laboratory, for which various steps of quality control (QC) are to be followed properly. In an automated clinical biochemistry lab, the autoanalyser uses various consumables like reagents, calibrators and QC materials for the routine testing of patient samples. The reagents used in the tests are the most consumed product in the laboratory. The amount of reagents consumed is determined by the total number of tests a laboratory receives, and the frequency of reagent kit change further depends on this. Among various analytes, plasma glucose is one of the analytes which is more frequently asked for and because of this, there will be a frequent reagent kit lot change of this analyte. A specific lot number is given to reagents of a particular batch which is manufactured using similar conditions [1]. The manufacturing conditions in which each of these lots is produced will be slightly different and it can lead to a slight change in the analytical process which is generally acceptable [2]. There is a chance that these changes can gradually build up over a period of time and affect the patient results without our knowledge [2]. In order to avoid these variations, proper measures have to be taken routinely, each time when a new reagent lot is introduced into a machine [3] [4]. As per the guidelines of CLSI (Clinical and Laboratory Standards Institute), laboratories should analyse and correct the changes in the performance of an analyte after the reagent lot change [5]. In Indian laboratories, National accreditation board for testing and calibration laboratories (NABL) is the agency which gives the certification and accreditation for the quality of the work they perform. According to NABL, the kit lot change should be accompanied by QC run, and the values of QC should not surpass the range set for the old lot [6].

During a reagent lot change, QC values with a new lot should fall within the same range (±2SD) set for the old lot [7]. This can be done with various numbers of levels of QC materials as per the lab's QC protocol. However, the QC material does not represent the patient sample, and in order to rule out the variation in this context, a number of patient samples should also be performed with the old and new reagent lots [3]. There is no definite international criterion which states the minimum sample size to be used for this purpose. What is more, this procedure varies from laboratory to laboratory in terms of the number of samples and the level of QC used. The reagents with a new lot number will be used when there is a consistency in these results based on the acceptance criteria put up by each lab [7]. Generally, reagents with a new lot will be accepted for use in a lab if the results obtained from different levels of QC material run using a new lot come within the ±2SD range of QC material set for the old reagent lot and if the variation between the results of the patient sample with the new lot and the old lot is less than 10 per cent [8]. If any of these criteria are not met, then the reagent lot change becomes invalid.

In this study, we have examined the variation in QC materials' results when compared to patient samples while a lot change of a reagent occurs. We have also analysed chances of long term drift in three analytes during five consecutive reagent lot changes in our laboratory.

## Materials and Methods

### Study design

The current study was performed in the clinical biochemistry laboratory of the Department of Biochemistry, Armed Forces Medical College, Pune, for a period of one year, from the 1^st^ of June 2018 to the 31^st^ of May 2019. All procedures performed in the studies involving human participants were in accordance with the ethical standards of the institutional research committee (Institutional ethics committee, AFMC, Pune, reference number - IEC/2019/292) and with the 1964 Helsinki declaration and its later amendments or comparable ethical standards. Informed consent was obtained from all individual participants included in the study. Our laboratory has been accredited by NABL for medical testing since 2010. For 9 years, we have been receiving re-accreditation based on our performance and quality of work in the laboratory. The last accreditation was given in February 2018 and it is valid until February 2020. The test results of patients and QC samples after 60 reagent lot change events were included in the analysis of this study. These reagent lot changes were performed in two Siemens Dimension EXL 200 auto analyzers and there were routine biochemistry analytes for which kit lot change was performed. The reagents used were purchased from the Siemens Company and the laboratory procedure for each analyte was followed according to the manufacturing company's instructions. The QC materials for Level 1 and 2 were purchased from Biorad (USA) and the mean and standard deviation was set according to the manufacturing company's instructions. Our laboratory validates the effect of change in the lot number of a reagent using the results of 10 patients' samples and QC materials with 2 levels (one with a normal and another with an abnormal range). The new reagent lot will be in use only when it meets the variation as per the cut-off values provided by Henry's textbook of clinical chemistry [9] [10]. For the patients' samples, the percentage of variation for each sample is obtained from the following equation: [(Patient result with the new lot - Patient result with the old lot)/Patient result with old lot] X 100. The results of QC are considered only when it falls within the range set for the old reagent lot. Our lab used to set the range for each analyte as mean ±2 SD from the data provided by the company. All the data were collected and double-checked for any variation. There was no QC material kit lot change during this period in our laboratory.

### Statistics

The results obtained from the patient samples and QC with the new and old lots during 60 reagent lot changes were first log-transformed. Both P (difference of patient sample) and ΔQC were calculated from the above data. In order to see the difference between the variation in QC and patient sample results, Wilcoxon sum rank test was performed in R 3.5.2 statistical software. Statistical significance was set at a P value less than 0.05. The analysis of DQC was performed to see the size and variation. The results of QC material with an old lot of a reagent were subtracted from a new lot of a reagent in order to get the DQC values. Then these DQC results were divided by their own SD values of each analyte which is used in monitoring the daily QC. This was expressed in terms of SD multiples. There were five classes of these values ranging from <0.5 to >2.0.

Next, the long-term effect of kit lot change for 3 analytes was analysed in which consecutive five reagent lot change data were available. Out of the 19 analytes which underwent reagent kit lot change, only three analytes had reagent lot change for 5 consecutive times during the study period of one year in our lab. This was due to the high demand for tests and a smaller number of tests per reagent lot. So only those were included in the long term drift analysis. The data of serum calcium, ALT and AST during their five consecutive reagent lot changes were collected. Using Weighted Deming Regression (WDR), regression coefficients and Jack-knife standard error were derived. WDR was performed using the mcr package of R 3.5.2 software. The intercept and slope of regression with a standard error during the change of reagent lots were analysed and t statistics was performed [7] in order to find out the cumulative shift in lot change.

## Results

In our study, practically none of the analytes which underwent kit lot change showed variation beyond acceptable limits as shown in Table 1. The P and QC results of 60 kit lot change results with patient samples and QC were analysed and it was found that 10 out of the 60 reagent lot changes (16.7%) differed significantly (P<0.05). These include albumin, phosphorus, calcium, total cholesterol, ALP, BUN, glucose, amylase and total protein. The complete data is given in Table 1. The kit lot change result for phosphorous was significantly different in two consecutive times. The results are grouped according to the nature of the method into pure chemical method results and enzymatic method results and presented in Table 1.

**Table 1:** Kit lot change events with differences between the results of patients’ samples and QC materials for various analytes (Wilcoxon Rank Sum test).

Analyte	Kit lot change date	P value	Analyte	Kit lot change date	P value
Chemical method (N=44)			Chemical method		
Albumin	01/06/2018	0.03	ALP	14/02/2019	0.36
Total protein	14/06/2018	0.76	Phosphorus	15/02/2019	0.48
Phosphorus	26/06/2018	0.03	Albumin	22/02/2019	0.11
Total bilirubin	26/06/2018	0.49	HDL	04/03/2019	0.61
Calcium	27/06/2018	0.61	Total protein	07/03/2019	0.03
ALP	27/06/2018	0.49	LDH	06/04/2019	0.12
ALT	12/07/2018	0.36	Creatinine	13/04/2019	0.49
Amylase	12/07/2018	0.49	Calcium	20/04/2019	0.12
Calcium	09/08/2018	0.03	ALT	29/04/2019	0.76
AST	11/08/2018	0.49	AST	11/05/2019	0.18
Creatinine	16/08/2018	0.61	Amylase	17/05/2019	0.03
CK	17/08/2018	0.18	TBI	21/05/2019	0.36
DBI	07/09/2018	1	Albumin	24/05/2019	0.59
LDH	11/09/2018	1	** Enzymatic method (N=16) **		
AST	11/09/2018	0.12	Triglycerides	01/08/2018	0.36
Calcium	25/09/2018	0.36	Uric acid	03/08/2018	0.76
Phosphorous	25/09/2018	0.03	BUN	17/08/2018	0.36
TBI	27/09/2018	0.61	Uric acid	28/09/2018	0.49
DBI	27/09/2018	0.12	Cholesterol	28/09/2018	0.03
ALP	23/10/2018	0.03	Glucose	28/09/2018	0.49
ALT	30/10/2018	0.18	Glucose	16/10/2018	0.27
ALT	06/11/2018	0.18	Triglycerides	06/11/2018	0.76
AST	16/11/2018	0.12	BUN	16/11/2018	0.03
ALT	24/12/2018	0.61	BUN	28/12/2018	0.61
Creatinine	27/12/2018	0.27	Cholesterol	15/02/2019	1
AST	07/01/2019	0.76	Uric acid	04/03/2019	0.18
LDH	10/01/2019	0.27	Triglycerides	05/04/2019	0.91
Calcium	16/01/2019	0.36	BUN	20/04/2019	0.18
TBI	13/02/2019	1	Glucose	29/04/2019	0.12
DBI	13/02/2019	0.12	Glucose	20/05/2019	0.03
CK	14/02/2019	0.48			

A total of 120 D_QC_ data were available with two levels of QC during 60 reagent lot change procedures. The sizes of these were determined by dividing them with SD set for each analyte. The D_QC_ size was represented as multiples of SD values. There were 4 D_QC_ results above 2SD range and they were for Albumin and BUN. The data are shown in Table 2.

**Table 2:** DQC size and percentage

Size (SD)	No.	%
<0.5	74	61.7
0.5–1.0	28	23.3
1.0–1.5	9	7.5
1.5–2.0	5	4.2
>2.0	4	3.3
**Total**	**120**	**100**

In order to evaluate the degree of bias between the results of two different lots of a reagent, regression analysis was performed. The regression analysis provides the values of a slope and an intercept which represent the degree of proportional and constant bias respectively. The data of the regression analysis of three analytes for 5 consecutive times are given in Table 3. In the regression analysis of calcium, there was a positive regression intercept and a positive regression slope in 3 reagent lot change events during June 2018 and May 2019. There were two negative regression intercepts with a positive regression slope. In the regression analysis of ALT, there was a positive regression intercept and a positive regression slope in 2 reagent lot change events during June 2018 and May 2019. There were three negative regression intercepts with a positive regression slope. In the regression analysis of AST, there was a positive regression intercept and a positive regression slope in the 2 reagent lot change events during June 2018 and May 2019. There were three negative regression intercepts with a positive regression slope. In regression analysis, the values of slopes were close to 1 in all three analytes which shows that there was no proportional bias in these analytes. But the values of intercepts in these cases were not close to 0 which shows the occurrence of a constant error in all the cases. This indicates the chances of a cumulative drift in the data of 3 analytes over a period of time.

**Table 3:** Regression slope and intercept derived using Weighed Deming Regression test with Jack-knife standard error for calcium, ALT and AST from June 2018 to May 2019

Calcium	Slope		Intercept	
** Date **	** Estimate **	** SE **	** Estimate **	** SE **
27/06/2018	0.976	0.049	0.222	0.447
09/08/2018	1.009	0.008	0.052	0.065
25/09/2018	1.072	0.108	-0.709	0.948
16/01/2019	1.002	0.042	0.012	0.396
20/04/2019	1.122	0.049	-1.008	0.440
** ALT **	** Slope **		** Intercept **	
** Date **	** Estimate **	** SE **	** Estimate **	** SE **
12/07/2018	1.053	0.068	-1.634	1.978
30/10/2018	0.952	0.062	1.227	1.697
06/11/2018	0.999	0.077	-0.079	2.056
24/12/2018	0.946	0.033	0.786	0.795
29/04/2019	0.999	0.026	-0.071	0.674
** AST **	** Slope **		** Intercept **	
** Date **	** Estimate **	** SE **	** Estimate **	** SE **
11/08/2018	1.048	0.059	-1.096	1.156
11/09/2018	1.008	0.027	-1.078	0.786
16/11/2018	1.010	0.013	0.296	0.268
07/01/2019	0.853	0.052	3.434	1.003
11/05/2019	1.027	0.040	-0.550	1.465

The t-statistics was performed for all 3 analytes, with the regression data and the results presented in Table 4. In the case of calcium, the t-statistic was less than critical t-value of proportional bias (1.66; 2.10) and constant bias (-1.45; 2.10) at 95% CI. So, there was no cumulative drift observed in serum calcium during this period. In the case of ALT, t-statistics was less than critical t-value of proportional bias (-0.37; 2.04) and constant bias (0.06; 2.05) at 95% CI. So, there was no cumulative drift observed in serum ALT during this period. In the case of AST, t-statistics was less than critical t-value of proportional bias (-0.60; 2.05) and constant bias (0.45; 2.05) at 95% CI. So, there was no cumulative drift observed in serum AST during this period.

**Table 4:** t statistics analysis for cumulative shift analysis of 3 analytes

Analyte	t statistic	Degrees of freedom	Critical t value	P-value	Conclusion
** Calcium **					
Slope analysis	1.66	17.71	2.10	0.12	No cumulative shift
Intercept analysis	-1.45	18.57	2.10	0.16	No cumulative shift
** ALT **					
Slope analysis	-0.37	28.73	2.04	0.71	No cumulative shift
Intercept analysis	0.06	27.86	2.05	0.95	No cumulative shift
** AST **					
Slope analysis	-0.60	26.81	2.05	0.55	No cumulative shift
Intercept analysis	0.45	27.54	2.05	0.66	No cumulative shift

## Discussion

Good laboratory practice consists of validating a new reagent lot before using it in the analyzer to ensure that patient sample results and QC results are consistent during the change in lots of reagents in a measurement procedure [9]. During kit lot change, a number of patient samples used to be run before and after the change of lot, as a part of routine quality assurance. These test results which were obtained were examined to verify the consistency between the results after the usage of each lot of reagents. Ideally, the set of patient samples used in this procedure should verify the consistency over the entire measuring interval of the analyte. Laboratory faces difficulty in getting the test samples which encompass the entire measuring interval; thus, patient samples in smaller intervals are taken to symbolize the consistency of consequences over the complete interval [9]. Preferably, when QC and the patients' test samples are measured with an old and new lot of reagent, their values are expected to be the same and this never happens. Therefore, every laboratory will have its own guidelines to check the consistency of QC and patient results during reagent lot change. General criteria for accepting a new reagent lot is that the result with a new kit falls within the predefined target range of ±2SD of QC material set for the old lot and the variation in the results of patient samples with both kits is less than 10% [8]. This criterion is not followed by most of the laboratories as these are not the mandatory guidelines given by the accreditation agencies.

The current study examined the variation of QC results when compared to the results of variation in patient sample results when there is a reagent lot change in the laboratory. Our data demonstrated that there was a 16.7% variation between the patient and QC results during the 60 reagent lot changes. It means that if we had done only the analysis with QC material, we would have missed the variation that had been identified with patient samples. In contrast, one previous research work by Cho et al. [11] showed that there was a significant difference in 7.8% of lot change in 360 reagent lot change events [11]. In another research paper, Miller et al. [9], presented that there was a variation in the 40.9% of events of reagent lot change In both studies, they had used two assay methods, general chemistry and immuno-assay, but, in our study we have included only general chemistry assays. Our laboratory uses a strategy in which we run two levels of QC along with 10 patients' samples when there is a reagent lot change. Previous studies used only five or fewer patients' samples during reagent lot change along with multiple levels of QC. We have used the Wilcoxon sum rank test for our analysis. The disparity in the results might be due to the variation in the level of QC and the number of samples tested during each lot change rather than due to the difference of the statistical method being used.

There are non-commutability problems when we use QC material to assess the impact of reagent lot change. This is because during the manufacturing, the matrix which is used for QC material making does not represent the patient sample. Therefore, using QC material result to validate the impact of reagent lot change is debatable [12] [13] [14]. For example, in one of the previous studies of alkaline phosphatase assay, the non-commutability problem was caused due to the usage of QC materials prepared from a non-human source [15]. Subsequently, the results obtained from QC material run during lot change event of a reagent are useful in adjusting the predefined range. It will minimize false negative or positive errors rates, which may further accumulate over some time. The false-negative and negative rates for particular QC material can be estimated after dividing D_QC_ results by SDs of corresponding analytes QC yielding multiples of SD [11].

As per our laboratory policy, the prede ned target range differs from the mean value by 2 SD, and there is a false detection rate of 3.3% during a reagent lot change which has a difference of SD more than 2 (Table 3). There were 120 DQC results available with two levels of QC during 60 events of lot change. After evaluating our DQC size, it was found that 96.7% QC results were inside the DQC size of 2. Thus, during 3.3% of lot change procedures, the range of QC should be readjusted. The occurrence of these non-commutable results with QC products is sufficient enough to prove that this alone should not be used to check the reliability of results when a reagent lot change occurs.

The current approaches used in clinical chemistry laboratory failed in detecting lot-to-lot change because the variation pointed out in this lot-to-lot change is small and neglectful. Moreover, if the lot-to-lot change remains undetected over time, it may accumulate to a significant level and become a longterm drift [2] [5]. A recent report has identified an undetected long-term drift during reagent lot change in Mayo Clinic [7]. Therefore, the long-term drift in reagent lots could be an important contributor to result in inconsistencies in clinical laboratories. To find out the long-term drift in reagent lots after consecutive reagent lot changes, we evaluated the results of the patient result data of three analytes using regression analysis. In the regression analysis, we couldn't find any cumulative shifts in any of the reagent lot changes in the analytes serum calcium, AST and ALT between June 2018 and May 2019. This shows that all three analytes we analysed were in the acceptable limits.

In conclusion, there was variability in QC results and patient results during reagent lot change in the 16.67% of analytes, suggesting that the use of QC material to find the impact of a new reagent lot doesn't have much utility in a clinical biochemistry lab. The difference in the values of patient samples is significant to validate a new lot of reagent, as there is a difference in values of QC material performed during kit lot change. It was found that there was no cumulative shift in the results of consecutive reagent lot change in our laboratory, and this may not be the case in all other laboratories. Hence, individual laboratories should verify their QC and patient results during reagent kit lot change, the step which is presently totally ignored by most of the laboratories.

## Acknowledgements

None.

## Conflict of interest statement

All the authors declare that they have no conflict of interest in this work.
